# CSDNet: A Novel Deep Learning Framework for Improved Cataract State Detection

**DOI:** 10.3390/diagnostics14100983

**Published:** 2024-05-08

**Authors:** Lahari P.L, Ramesh Vaddi, Mahmoud O. Elish, Venkateswarlu Gonuguntla, Siva Sankar Yellampalli

**Affiliations:** 1 Department of Electronics and Communication Engineering, SRM University AP, Andhra Pradesh, India; lahari_p@srmap.edu.in (L.P.); ramesh.v@srmap.edu.in (R.V.); 2 Computer Science Department, Gulf University for Science and Technology, Hawally 32093, Kuwait; elish.m@gust.edu.kw; 3 GUST Engineering and Applied Innovation Research Center, Gulf University for Science and Technology, Hawally 32093, Kuwait; 4 Symbiosis Centre for Medical Image Analysis, Symbiosis International (Deemed University), Pune, India

**Keywords:** cataract, visual impairment, pre-trained convolutional neural networks, classification, detection

## Abstract

Cataracts, known for lens clouding and being a common cause of visual impairment, persist as a primary contributor to vision loss and blindness, presenting notable diagnostic and prognostic challenges. This work presents a novel framework called the Cataract States Detection Network (CSDNet), which utilizes deep learning methods to improve the detection of cataract states. The aim is to create a framework that is more lightweight and adaptable for use in environments or devices with limited memory or storage capacity. This involves reducing the number of trainable parameters while still allowing for effective learning of representations from data. Additionally, the framework is designed to be suitable for real-time or near-real-time applications where rapid inference is essential. This study utilizes cataract and normal images from the Ocular Disease Intelligent Recognition (ODIR) database. The suggested model employs smaller kernels, fewer training parameters, and layers to efficiently decrease the number of trainable parameters, thereby lowering computational costs and average running time compared to other pre-trained models such as VGG19, ResNet50, DenseNet201, MIRNet, Inception V3, Xception, and Efficient net B0. The experimental results illustrate that the proposed approach achieves a binary classification accuracy of 97.24% (normal or cataract) and an average cataract state detection accuracy of 98.17% (normal, grade 1—minimal cloudiness, grade 2—immature cataract, grade 3—mature cataract, and grade 4—hyper mature cataract), competing with state-of-the-art cataract detection methods. The resulting model is lightweight at 17 MB and has fewer trainable parameters (175, 617), making it suitable for deployment in environments or devices with constrained memory or storage capacity. With a runtime of 212 ms, it is well-suited for real-time or near-real-time applications requiring rapid inference.

## 1. Introduction

The eye is prone to various disorders, notably cataracts, causing vision loss if untreated [1,2]. Factors like aging, diabetes, UV radiation, genetics, pollutants, eye trauma, and habits contribute to cataracts [3]. Diagnosis involves a comprehensive eye exam, and treatment includes removing the clouded lens and implanting an artificial one. Cataracts are graded based on cloudiness and vision impact, with stages as follows: grade 1, characterized by an incipient cataract with minimal vision impact; grade 2, an immature cataract featuring noticeable cloudiness and slight vision blurring; grade 3, a mature cataract significantly impairing vision, particularly at night; and grade 4, a hyper mature cataract, exhibiting lens changes, reduced vision, and potential complications such as glaucoma.

Deep learning has revolutionized eye medical imaging across various tasks [4,5,6,7,8,9], automating cataract detection and classification for precise diagnosis [9]. It aids in diabetic retinopathy screening, identifying severity for timely intervention [10], and enables early glaucoma detection through optic nerve analysis [11]. Deep learning segments retinal vessels and optic discs, assisting in vascular change analysis and glaucoma diagnosis [12]. It identifies age-related macular degeneration early and monitors eye conditions via OCT [13], analyzes corneal diseases through corneal images [14], and guides surgeries like cataract surgery precisely [15]. In telemedicine, it monitors changes for timely intervention and facilitates algorithm development through synthetic image generation [16]. 

Enhanced cataract state classification and detection are crucial for precise diagnosis and treatment, potentially improving patient outcomes and healthcare efficiency. Convolutional neural networks (CNNs) are commonly used for image classification and detection, benefiting from data preparation, model selection, and augmentation for improved generalization [17,18]. 

Several deep learning methods in the literature aim to advance cataract state detection, as outlined below. In ref. [19], the authors achieve a high training accuracy of 99.47% and validation accuracy of 97.94% using NasNet Mobile for cataract classification. Ref. [20] categorizes cataracts into normal and cataract groups, with average accuracies ranging from 91.06% to 93.50% using models like VGG19 and ResNet. Transfer learning is applied in [21], where an ensemble technique combining VGG19, ResNet101V2, and InceptionV3 achieves an F1_score of 95.90% on a test dataset. In ref. [22], the EYENET model, serving as a self-diagnosis tool for five eye disorders, achieves an accuracy of 92.3%, potentially alleviating doctor burden and enabling the rapid detection of ailments. In the literature, several pre-trained CNN models such as VGG [23], ResNet [24], DenseNet [25], Inception [26], MIRnet [27], Xception [28,29], and EfficientNet [30] have been utilized to train large image datasets, as outlined in Table 1.

However, efforts to improve cataract classification and detection have faced challenges in detecting early stages accurately, reducing overall accuracy. Variations in image quality and patient characteristics further complicate classification. Simpler models may face challenges in understanding complex data patterns, which can lead to lower performance in tasks requiring intricate relationships. Underfitting becomes a concern when the model fails to represent the data patterns effectively. Nonetheless, simpler models offer advantages in memory-constrained environments, with fewer parameters yet still-capable representation learning. This feature renders them suitable for real-time applications where rapid inference is essential.

**Figure 1:**
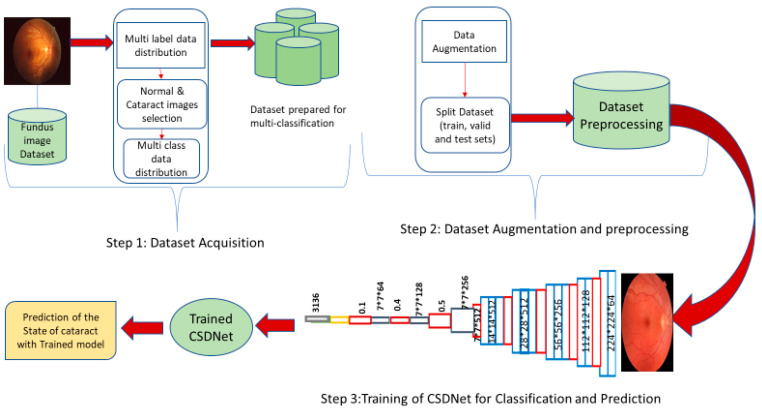
The proposed framework of improved cataract state classification.

The depicted model in Figure 1 introduces an enhanced framework of improved cataract state classification, addressing the aforementioned issues in detecting cataracts and their severity by utilizing a combination of convolutional layers, dense layers, and dropout layers. The framework comprises three main stages: dataset preparation for binary (normal or cataract) and multi-class (normal, grade 1—minimal cloudiness, grade 2—immature cataract, grade 3—mature cataract, and grade 4—hyper mature cataract) classification, data augmentation and preprocessing, and the Cataract States Detection Network (CSDNet) for the precise classification and detection of cataract states.

This proposed architecture enhances the model’s capability to discern intricate patterns within data, capturing subtle cataract indications and accurately identifying cataracts while predicting their severity. It achieves this by learning stage-specific features, thus enabling the categorization of cataract progression from early to severe stages. Evaluation metrics such as accuracy, precision, F1_score, and recall are employed for cataract state classification. Furthermore, the model is designed to be lightweight and suitable for deployment in memory-constrained environments or devices with limited storage capacity, maintaining a reasonable number of parameters for learning from data while being efficient in memory consumption and computational resources. These attributes enable the model to excel in tasks like image recognition and classification, rendering it suitable for real-time applications. 

The remainder of this paper is organized as follows: The next section provides a detailed explanation of the proposed model for enhanced classification and detection, while Section 3 presents the detailed results and metrics evaluation. Section 4 offers detailed discussions and conclusions of the paper. 

## 2. Proposed Model

The CSDNet model is designed for efficient deployment on memory-constrained devices, featuring tailored customization for enhanced detection capabilities. It employs global average pooling and reshaping layers to capture essential global features, while dropout regularization prevents overfitting and ReLu activation functions aid convergence. 

The proposed model consists of 14 layers, including a flattened layer and a sigmoid activation function, providing a balance between accuracy and complexity for efficient deployment. The input layer is optimized for 224 × 224 images with three channels (RGB). Functional, dense, and dropout layers are integrated, leveraging pre-trained models for feature extraction. Functional layers integrate pre-trained models or complex sub-networks into the architecture, enabling the reuse of established architectures as building blocks. Dense layers, also known as fully connected layers, perform linear transformations on input data followed by non-linear activation functions, connecting each neuron to every neuron in the previous layer. Dropout layers prevent overfitting by randomly deactivating neurons during training, encouraging the learning of robust features and improving generalization. Activation functions introduce non-linearity to the model. They are, in general, employed to acquire high-level representations and patterns within the data. Typically found in the hidden layers of feedforward neural networks, they play a common role in capturing intricate features.

**Figure 2:**
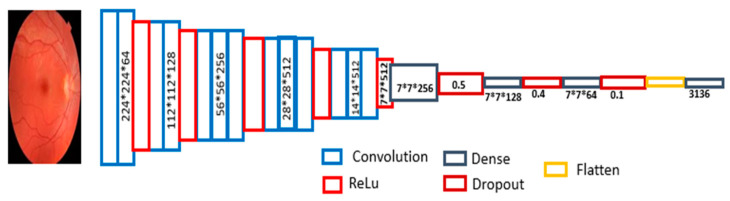
CSDNet architecture.

The model, as illustrated in Figure 2, begins by passing the input through a functional layer for feature extraction. The number of filters in a CNN is crucial and influenced by multiple factors. More filters enhance the model’s capacity to detect intricate patterns, beneficial for tasks needing fine detail for accurate detection. Moreover, employing more filters helps the network learn diverse feature representations per layer, facilitating the extraction of hierarchical features necessary for understanding input data. Starting with a larger number of filters allows the network to capture various low-level features initially, which are then abstracted into higher-level features in subsequent layers.

To select the optimal model configuration, a series of experiments were conducted, varying the number of blocks and filters (presented in the results section as Table 2. Model accuracy with various combinations of blocks and filters). The findings revealed that the most effective model consists of four blocks with varying filter sizes (64, 128, 256, 512) and is the proposed model. The proposed model comprises four convolutional layers, with ascending filter dimensions of 64, 128, 256, and 512, respectively. It accepts input of shape (224, 224, 3), with each convolutional layer followed by ReLU activation to maintain spatial information. Consequent to the convolutional layers, a functional layer executes convolution operations, resulting in an output shape of (7, 7, 512). Following this, three dense layers are integrated, with decreasing neuron quantities: 256, 128, and 64. Each dense layer is paired with a dropout layer (with dropout rates of 0.5, 0.2, and 0.1, respectively) to mitigate overfitting, resulting in an output (7, 7, 64). This output is then processed through a flattened layer, converting the previous output shape (7, 7, 64) into a one-dimensional array of size 3136. Finally, a dense layer with sigmoid activation, suitable for binary classification tasks, is employed. For the detection of cataract states, softmax is utilized.

## 3. Results and Discussion

In this section, a detailed description of the dataset chosen, data preparation, implementation details, evaluation metrics, and results of the proposed model in comparison with the existing models are discussed in detail.

Data: This study uses the Ocular Disease Intelligent Recognition (ODIR) database [31], containing structured ophthalmic data from 5000 patients, including age details and color fundus images of both eyes. Each patient record includes diagnostic keywords provided by medical professionals. The database mirrors real-world patient data collected from various hospitals and medical facilities across China by Shanggong Medical Technology Co., Ltd. Fundus images exhibit resolution variations due to different camera usage. Trained human readers meticulously annotated the dataset to ensure quality control. Patients are classified into 8 distinct categories: Normal, Diabetes, Glaucoma, Cataract, Age-related macular degeneration, Hypertension, Pathological myopia, and other diseases/abnormalities. 

As the emphasis is on enhancing cataract state classification and detection, only categories Cataract (C, 1168 images) and Normal (N, 1000 images), 2168 images in total, were chosen for further analysis. On the removal of the noisy and blurry images—data cleaning, the total number was images is 2000 (C—1100 (grade 1—230; grade 2—137; grade 3—469; grade 4—264) and N—900 images). 

Preprocessing: The preprocessing procedures involve the following steps: Image resizing, which standardizes all images to a uniform size to reduce computational complexity and ensure compatibility with the model’s architecture. Normalization, which adjusts pixel values of the images to a standardized range (typically 0 to 1) to stabilize the training process and potentially improve model convergence. Finally, data augmentation encompasses rotating, flipping, and shifting images to produce supplementary samples, thereby enlarging the dataset. As a result of the data augmentation, the dataset is expanded to 8000 images and is included in further analysis. 

Data for training, testing, and validation: The preprocessed dataset comprising 8000 color fundus images, reflecting diverse eye conditions and demographic profiles, underwent a meticulous process of preparation and partitioning for training, testing, and validation. Through label encoding, diagnostic terms were numerically represented, with 0 denoting normal eyes and 1 indicating the presence of cataracts in binary classification. In this analysis, we explored different splitting strategies (90/05/05; 80/10/10; 70/15/15; 60/20/20) to assess classification accuracy. For instance, following an 80/10/10 split strategy, the dataset was divided, allocating 80% (6400 images) for training, 10% (800 images) for testing, and another 10% (800 images) for validation purposes. 

Implementation details: All experiments were conducted on a computer with the following properties: Intel(R) core i3 processor, 7th Generation, and 8 GB RAM. We utilized Google Collab with a T4 GPU to accelerate our model training and experiments, resulting in faster processing. Preprocessing, augmentation, VGG19, ResNet 50, DenseNet 201, inception v3, MIRNet, Xception, and Efficient net B0 models were implemented using Python (Google Collab), Keras environment. The proposed model was optimized using Adam Optimizer with a learning rate of 0.001, and a batch size of 15 for memory efficiency. 

Evaluation criteria: In this paper, accuracy, recall (also known as the sensitivity or true positive rate), precision (the model’s ability to properly identify positive cases), and F1_score (harmonic mean of precision and recall to provide a balanced assessment of a model’s performance) are the metrics used as evaluation indicators. They are defined as follows:

(1) $$Accuracy=\frac{TP+TN}{TP+TN+FP+FN}$$

(2) $$Recall=\frac{TP}{TP+FN}$$

(3) $$Precision=\frac{TP}{TP+FP}$$

(4) $$F1\_score=\frac{2*(Precision*Recall)}{\left(Precision+Recall\right)}$$

where TP is true positive, TN is true negative, FP is false positive, and FN is false negative.

Performance of the proposed model: In this section, we extensively analyze the performance of the proposed model. We evaluate our model alongside eight other pre-trained models for cataract classification and detection using identical datasets. We compare the experimental outcomes with state-of-the-art methods for cataract classification and detection.

First, to select the most suitable model configuration, a series of experiments were conducted using an 80/10/10 split strategy, varying the number of blocks and filters, which are tabulated in Table 2. The results show that the most effective model consists of four blocks with varying filter sizes (64, 128, 256, 512) and is the proposed model.

The proposed CSDNet, along with several pre-trained CNN models such as VGG19, ResNet50, DenseNet201, InceptionV3, MIRNet, Xception, and EfficientNetB0, were developed and tested for binary classification (cataract or normal) using a dataset comprising 8000 images (4400 cataract and 3600 normal) after preprocessing. The evaluation metrics and accuracies of all models were compared and are tabulated in Table 3—metrics comparison of existing and proposed model for the classification of cataract and normal data. Various training, testing, and validation set combinations (90/05/05; 80/10/10; 70/15/15; 60/20/20) were employed. The results indicate that the 80-10-10 split ratio consistently yielded better accuracy, precision, recall, and F1_score across most models, including the proposed CSDNet, thus becoming the chosen splitting strategy.

With the chosen model and split strategy, the cataract states were now classified using the same model, transitioning from binary classification to multi-class classification (replacing sigmoid with softmax). Each class is represented by the following number of preprocessed images: grade 1—920; grade 2—548; grade 3—1876; grade 4—1056; and Normal (N)—3600. To address class imbalance, 548 images per class (totaling 2740 images) were randomly selected, given that grade 2 had only 548 images after preprocessing. Using the 80-10-10 split ratio, the evaluation metrics and accuracies for all models, including the proposed CSDNet, in the cataract state detection task were tabulated in Table 4—metrics comparison of existing and proposed model for cataract state detection. The results indicate that the proposed CSDNet outperformed others, achieving an average cataract state detection accuracy of 98.17%.

The collective experimental findings demonstrate that the proposed method attained a binary classification accuracy of 97.24% (distinguishing normal from cataract) and an average cataract state detection accuracy of 98.17% (encompassing normal, as well as grades 1 through 4 of cataract severity). This performance matches top cataract detection methods and consistently surpasses other models in all evaluation aspects.

To assess efficiency in terms of model size, layer count, and average running time, all pretrained models were compared alongside the proposed model, and the results were tabulated in Table 5—model comparison of existing and proposed models. The findings highlight several advantages of the proposed model: 1. Model Size—it has a relatively smaller size compared to most other models listed, with only 17 MB. This compactness renders it more lightweight and adaptable for deployment in memory-limited environments or devices with constrained storage capacity; 2. Trainable parameters—despite having fewer trainable parameters than most models, it maintains a reasonable count of parameters (1, 75, 617), enabling effective learning from data; 3. Average run time—with an average run time of 212 ms, it is well-suited for real-time or near-real-time applications requiring quick inference. These characteristics make the proposed model efficient in terms of both memory consumption and computational resources while still maintaining good performance in various tasks like image recognition, classification, or other related tasks.

**Figure 3:**
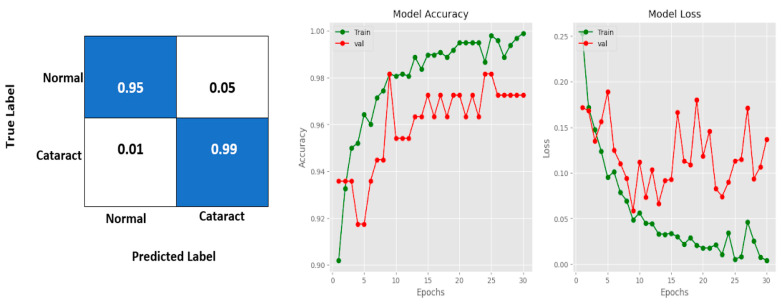
Confusion matrix, model accuracy, and model loss plots using the CSDNet for binary classification.

**Figure 4:**
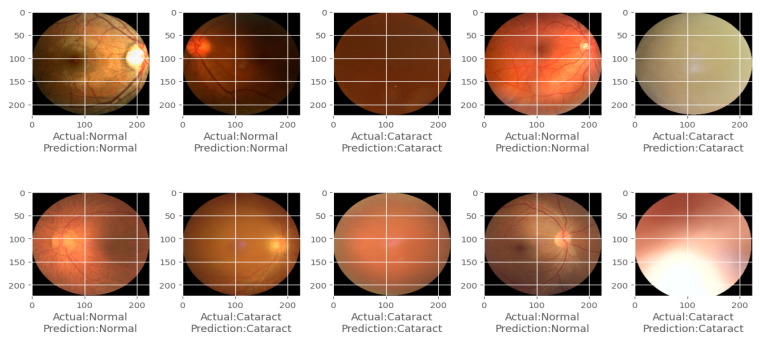
Actual and predicted classification of normal and cataracts using the CSDNet for binary classification.

The confusion matrix, model accuracy, and model loss plots obtained by using the CSDNet for binary classification are depicted in Figure 3. Additionally, Figure 4 provides an example of actual versus predicted outcomes for normal and cataract classifications using the CSDNet. The training and validation accuracy are plotted over 30 epochs, displaying the model’s training progress alongside validation results for accuracy and loss metrics across epochs. The proposed model achieved a classification accuracy of 97.24% and a loss of 0.1368.

**Figure 5:**
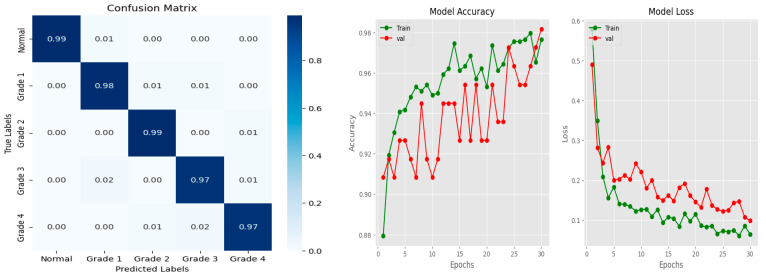
Confusion matrix, model accuracy, and model loss plots using the CSDNet for cataract state detection.

**Figure 6:**
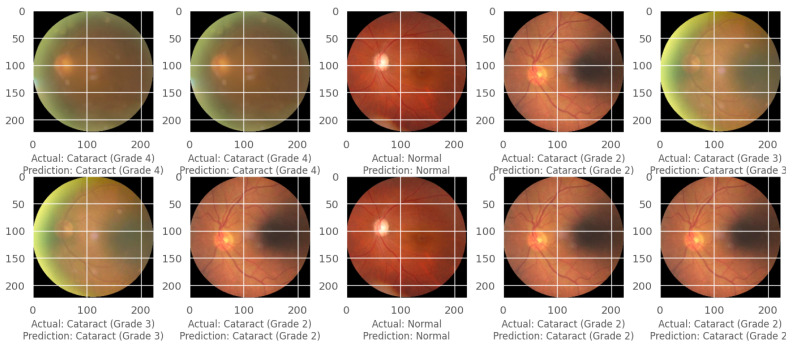
Actual and detected cataract states using the CSDNet for cataract state detection.

Similarly, the confusion matrix, model accuracy, and model loss plots for cataract state detection using the CSDNet are presented in Figure 5. Furthermore, Figure 6 showcases an instance of actual versus predicted states for normal and cataract conditions employing the CSDNet. The training and validation accuracy are illustrated over 30 epochs, with the curve representing the model’s training and validation performance concerning accuracy and loss metrics across epochs. The proposed model achieved an accuracy of 98.17%, with a loss of 0.0983.

## 4. Conclusions

In this study, we proposed a framework for classifying cataracts and detecting their states. Our proposed CSDNet achieved an accuracy of 98.17% in detecting cataract states. Comparison with pre-trained models revealed that the proposed method has improved the accuracy of cataract state detection. Furthermore, we compared our proposed CSDNet for cataract state detection with existing models and summarized the results in Table 6. The findings indicate that our model either matches or surpasses existing ones. Additionally, the lightweight nature of the CSDNet, requiring only 17 MB of memory, significantly lowers the barriers to deploying advanced diagnostic tools in low-resource settings. With a rapid inference time of 212 ms, the CSDNet holds promise for real-time diagnostic capabilities. Its high detection accuracy despite limited trainable parameters illustrates the feasibility of achieving precise diagnostics without extensive computational resources. Future work will focus on real-time implementation for the detection of cataracts and its evolution into a portable kit aimed at early cataract detection. 

## Figures and Tables

**Table 1:** Pre-trained CNN models for large image datasets (ALs—architecture layers; P—parameters (millions); INT1A—ImageNet Top-1 Accuracy (%); INT5A—ImageNet Top-5 Accuracy (%); CE—computational efficiency; R—reliability; PE—power efficiency; I—interpretability; TL—transfer learning; DA—data augmentation; HPs—hyper parameters; RTs—regularization techniques; L—low; M—moderate; H—high; NS—not specified).

Model	ALs	P	INT1A	INT5A	CE	R	PE	I	TL	DA	HPs	RTs
VGG19 [23]	19	143.67	~71	~89	L	M	L	H	Yes	Yes	Yes	Yes
ResNet 50 [24]	50	25.6	~76	~93	M	H	M	M	Yes	Yes	Yes	Yes
DenseNet 201 [25]	201	20.0	~77	~93	M	H	M	M	Yes	Yes	Yes	Yes
Inception V3 [26]	Variable	23.8	~77	~93	M	M	M	H	Yes	Yes	Yes	Yes
MIRNet [27]	Variable	NS	NS	NS	M	M	M	H	Yes	Yes	Yes	Yes
Xception [28,29]	Variable	22.9	~79	~94	M	H	M	H	Yes	Yes	Yes	Yes
EfficientNet B0 [30]	20	5.3	~77	~93	H	H	H	M	Yes	Yes	Yes	Yes

**Table 2:** Model accuracy with various combinations of blocks and filters.

Blocks	Filter Set	Accuracy
3	16, 32, 64	90.56%
4	32, 64, 128, 256	95.41%
4	64, 128, 256, 512	97.24%
5	32, 64, 128, 256, 512	94.03%

**Table 3:** Metrics comparison of existing and proposed models for the classification of cataract and normal data.

Normal vs. Cataracts	Model	Accuracy	Precision	Recall	F1_Score
Training set (90%), testing set (5%), and validation set (5%)	Vgg19	95.41	0.94	0.97	0.96
	ResNet 50	94.03	0.97	0.92	0.95
	DenseNet 201	93.11	0.92	0.95	0.94
	InceptionV3	94.03	0.93	0.96	0.94
	MIRNet	95.41	0.98	0.94	0.96
	Xception	95.41	0.94	0.98	0.96
	EfficientNet B0	95.87	0.97	0.94	0.96
	CSDNet	96.79	0.98	0.97	0.97
Training set (80%), testing set (10%), and validation set (10%)	Vgg19	95.87	0.95	0.97	0.96
	ResNet 50	97.24	1.00	0.95	0.97
	DenseNet 201	90.82	0.86	1.00	0.93
	InceptionV3	97.24	0.95	1.00	0.98
	MIRNet	94.50	0.98	0.91	0.94
	Xception	91.74	0.91	0.95	0.93
	EfficientNet B0	98.16	0.97	1.00	0.98
	CSDNet	97.24	0.97	0.99	0.98
Training set (70%), testing set (15%), and validation set (15%)	Vgg19	92.04	0.92	0.92	0.92
	ResNet 50	92.66	0.90	0.96	0.93
	DenseNet 201	95.10	0.96	0.96	0.96
	InceptionV3	95.72	0.93	0.99	0.96
	MIRNet	92.66	0.95	0.91	0.93
	Xception	91.13	0.93	0.90	0.91
	EfficientNet B0	95.71	0.95	0.97	0.96
	CSDNet	92.35	0.89	0.98	0.93
Training set (60%), testing set (20%), and validation set (20%)	Vgg19	95.07	0.95	0.97	0.96
	ResNet 50	94.03	0.94	0.95	0.94
	DenseNet 201	94.27	0.93	0.98	0.95
	InceptionV3	92.43	0.95	0.91	0.93
	MIRNet	93.81	0.95	0.93	0.94
	Xception	87.61	0.88	0.89	0.88
	EfficientNet B0	94.18	0.94	0.95	0.95
	CSDNet	95.18	0.94	0.97	0.96

**Table 4:** Metrics comparison of existing and proposed models for cataract state detection.

Normal, Grade 1 to 4	Model	Accuracy	Precision	Recall	F1_Score
Training set (80%), testing set (10%), and validation set (10%)	Vgg19	97.04	Normal:0.98 Grade 1:0.96 Grade 2:0.96 Grade 3.0.96 Grade 4:0.99	0.99 0.96 0.97 0.96 0.97	0.99 0.96 0.97 0.96 0.98
	ResNet 50	96.81	Normal:0.99 Grade 1:0.97 Grade 2:0.95 Grade 3.0.96 Grade 4:0.97	0.98 0.97 0.98 0.95 0.96	0.98 0.97 0.97 0.95 0.96
	DenseNet 201	90.74	Normal:0.95 Grade 1:0.87 Grade 2:0.88 Grade 3:0.90 Grade 4:0.94	0.93 0.90 0.91 0.89 0.90	0.94 0.89 0.89 0.89 0.92
	InceptionV3	96.17	Normal:0.98 Grade 1:0.98 Grade 2:0.93 Grade 3:0.95 Grade 4:0.97	0.97 0.96 0.98 0.95 0.95	0.97 0.97 0.96 0.95 0.96
	MIRNet	93.54	Normal:0.97 Grade 1:0.91 Grade 2:0.92 Grade 3:0.93 Grade 4:0.95	0.95 0.95 0.96 0.90 0.92	0.96 0.93 0.94 0.91 0.93
	Xception	91.23	Normal:0.93 Grade 1:0.91 Grade 2:0.88 Grade 3:0.90 Grade 4:0.95	0.94 0.90 0.91 0.90 0.91	0.94 0.90 0.89 0.90 0.93
	EfficientNet B0	97.81	Normal:0.98 Grade 1:0.97 Grade 2:0.99 Grade 3.0.95 Grade 4:0.98	0.98 0.96 0.99 0.97 0.97	0.98 0.97 0.99 0.96 0.97
	CSDNet	98.17	Normal:1.00 Grade 1:0.97 Grade 2:0.98 Grade 3.0.97 Grade 4:0.98	0.99 0.98 0.99 0.97 0.97	0.99 0.98 0.99 0.97 0.97

**Table 5:** Model comparison of existing and proposed models.

Model	Layers	Trainable Parameters	Model Size	Average Run Time
VGG19	19	200, 49, 473	574 MB	267 ms
ResNet 50	50	3, 01, 057	98 MB	118 ms
DenseNet 201	201	488, 85, 505	80 MB	185 ms
MIRnet	45	3, 93, 217	128 MB	276 ms
Inception V3	48	51, 201	92 MB	226 ms
Xception	71	10, 49, 601	88 MB	207 ms
EfficientNet B0	214	6, 56, 385	29 MB	291 ms
CSDNet	14	1, 75, 617	17 MB	212 ms

**Table 6:** Accuracy comparisons of existing and proposed models for cataract state detection.

Model	Dataset	Accuracy
Cataract detection using deep learning [3]	Mixed databases and internet images	92.7%
Early cataract detection using deep learning [10]	1600 images	93.10%
Computer-aided cataract severity diagnosis using pre-trained CNNs for feature extraction [11]	Online platforms	96%
CSDNet—proposed model with fewer layers and runtime	Open-source dataset [31]	98.17%

## Data Availability

The dataset utilized in this research was obtained from a publicly accessible source. Access to the dataset can be obtained via the reference provided in this paper. All other findings made in this study are fully detailed within the paper itself.

## References

[1] Pratap T., Kokil P. (2019). Computer-Aided Diagnosis of Cataract Using Deep Transfer Learning. Biomed. Signal Process. Control.

[2] Xu X., Zhang L., Li J., Guan Y., Zhang L. (2019). A Hybrid Global-Local Representation CNN Model for Automatic Cataract Grading. IEEE J. Biomed. Health Inform..

[3] Varma N., Yadav S., Kant J. (2023). A Reliable Automatic Cataract Detection Using Deep Learning. Int. J. Syst. Assur. Eng. Manag..

[4] Ting D.S.W., Pasquale L.R., Peng L., Campbell J.P., Lee A.Y., Raman R., Tan G.S.W., Schmetterer L., Keane P.A., Wong T.Y. (2018). Artificial Intelligence and Deep Learning in Ophthalmology. Br. J. Ophthalmol..

[5] Tong Y., Lu W., Yu Y., Shen Y. (2020). Application of Machine Learning in Ophthalmic Imaging Modalities. Eye Vis..

[6] Balyen L., Peto T. (2019). Promising Artificial Intelligence-Machine Learning-Deep Learning Algorithms in Ophthalmology. Asia-Pac. J. Ophthalmol..

[7] Razzak M.I., Naz S., Zaib A. (2017). Deep Learning for Medical Image Processing: Overview, challenges and the future. Lect. Notes Comput. Vis. Biomech..

[8] Lv Q., Zhang S., Wang Y. (2022). Deep Learning Model of Image Classification Using Machine Learning. Adv. Multimed..

[9] Junayed M.S., Islam M.B., Sadeghzadeh A., Rahman S. (2021). CataractNet: An Automated Cataract Detection System Using Deep Learning for Fundus Images. IEEE Access.

[10] Yadav S., Kant J. (2023). Automatic Cataract Severity Detection and Grading Using Deep Learning. J. Sens..

[11] Yadav J.K.P.S., Yadav S. (2022). Computer-Aided Diagnosis of Cataract Severity Using Retinal Fundus Images and Deep Learning. Comput. Intell..

[12] Akter N., Fletcher J., Perry S., Simunovic M.P., Briggs N., Roy M. (2022). Glaucoma Diagnosis Using Multi-Feature Analysis and a Deep Learning Technique. Sci. Rep..

[13] Ran A.R., Tham C.C., Chan P.P., Cheng C.-Y., Tham Y.-C., Rim T.H., Cheung C.Y. (2020). Deep Learning in Glaucoma with Optical Coherence Tomography: A Review. Eye.

[14] Keenan T.D.L., Chen Q., Agrón E., Tham Y.-C., Goh J.H.L., Lei X., Ng Y.P., Liu Y., Xu X., Cheng C.-Y. (2022). DeepLensNet: Deep Learning Automated Diagnosis and Quantitative Classification of Cataract Type and Severity. Ophthalmology.

[15] Tham Y.-C., Goh J.H.L., Anees A., Lei X., Rim T.H., Chee M.-L., Wang Y.X., Jonas J.B., Thakur S., Teo Z.L. (2022). Detecting Visually Significant Cataract Using Retinal Photograph-Based Deep Learning. Nat. Aging.

[16] Schünke L.C., Mello B., da Costa C.A., Antunes R.S., Rigo S.J., de Oliveira Ramos G., da Rosa Righi R., Scherer J.N., Donida B. (2022). A Rapid Review of Machine Learning Approaches for Telemedicine in the Scope of COVID-19. Artif. Intell. Med..

[17] Rawat W., Wang Z. (2017). Deep Convolutional Neural Networks for Image Classification: A Comprehensive Review. Neural. Comput..

[18] Valente J., António J., Mora C., Jardim S. (2023). Developments in Image Processing Using Deep Learning and Reinforcement Learning. J. Imaging.

[19] El-Baz A. (2018). Classification of Retinal Diseases Based on OCT Images. Front. Biosci..

[20] Thakur K., Kaur M., Kumar Y. (2023). A Comprehensive Analysis of Deep Learning-Based Approaches for Prediction and Prognosis of Infectious Diseases. Arch. Comput. Methods Eng..

[21] Kora P., Ooi C.P., Faust O., Raghavendra U., Gudigar A., Chan W.Y., Meenakshi K., Swaraja K., Plawiak P., Rajendra Acharya U. (2022). Transfer Learning Techniques for Medical Image Analysis: A Review. Biocybern. Biomed. Eng..

[22] Liesegang T.J., Hoskins H.D., Albert D.M., O’Day D.H., Spivey B.E., Sadun A.A., Parke D.W., Mondino B.J. (2003). Ophthalmic Education: Where Have We Come From, and Where Are We Going?. Am. J. Ophthalmol..

[23] Very Deep Convolutional Networks for Large-Scale Image Recognition. ar5iv. https://ar5iv.labs.arxiv.org/html/1409.1556.

[24] Yu L., Chen H., Dou Q., Qin J., Heng P.-A. (2017). Automated Melanoma Recognition in Dermoscopy Images via Very Deep Residual Networks. IEEE Trans. Med. Imaging.

[25] Lodhi B., Kang J. (2019). Multipath-DenseNet: A Supervised Ensemble Architecture of Densely Connected Convolutional Networks. Inf. Sci..

[26] Szegedy C., Vanhoucke V., Ioffe S., Shlens J., Wojna Z. Rethinking the Inception Architecture for Computer Vision. Proceedings of the 2016 IEEE Conference on Computer Vision and Pattern Recognition (CVPR).

[27] Zhang Z., Li G., Wang L. (2022). Seismic Random Noise Suppression Based on Mirnet with Dense Feature Fusion. IEEE Geosci. Remote Sens. Lett..

[28] Chollet F. Xception: Deep Learning with Depthwise Separable Convolutions. Proceedings of the 2017 IEEE Conference on Computer Vision and Pattern Recognition (CVPR).

[29] Chen L., Li S., Bai Q., Yang J., Jiang S., Miao Y. (2021). Review of Image Classification Algorithms Based on Convolutional Neural Networks. Remote Sens..

[30] Zhou A., Ma Y., Ji W., Zong M., Yang P., Wu M., Liu M. (2022). Multi-Head Attention-Based Two-Stream EfficientNet for Action Recognition. Multimed. Syst..

[31] https://www.kaggle.com/code/matthewmaddock/ocular-disease-recognition-model-training/input.

